# Characterization of novel bacteriophage phiC119 capable of lysing multidrug-resistant Shiga toxin-producing *Escherichia coli* O157:H7

**DOI:** 10.7717/peerj.2423

**Published:** 2016-09-13

**Authors:** Luis Amarillas, Cristóbal Chaidez, Arturo González-Robles, Yadira Lugo-Melchor, Josefina León-Félix

**Affiliations:** 1 Laboratorio de Biología Molecular y Genómica Funcional, Centro de Investigación en Alimentación y Desarrollo, A. C., Culiacán, Sinaloa, México; 2 Laboratorio de Genética, Instituto de Investigación Lightbourn, A. C., Cd. Jiménez, Chihuahua, México; 3 Inocuidad Alimentaria, Centro de Investigación en Alimentación y Desarrollo, A. C., Culiacán, Sinaloa, México; 4 Departamento de Infectómica y Patogénesis Molecular, Centro de Investigación y de Estudios Avanzados, Instituto Politécnico Nacional, Ciudad de México, México; 5 Laboratorio de Biología Molecular de la Unidad de Servicios Analíticos y Metrológicos, Centro de Investigación y Asistencia en Tecnología y Diseño del Estado de Jalisco A. C., Guadalajara, Jalisco, México

**Keywords:** Shiga toxin, Phage phiC119, Genome analysis, *Siphoviridae*, Biocontrol applications, Phage group relationships

## Abstract

**Background:**

Shiga toxin-producing *Escherichia coli* (STEC) is one of the most common and widely distributed foodborne pathogens that has been frequently implicated in gastrointestinal and urinary tract infections. Moreover, high rates of multiple antibiotic-resistant *E. coli* strains have been reported worldwide. Due to the emergence of antibiotic-resistant strains, bacteriophages are considered an attractive alternative to biocontrol pathogenic bacteria. Characterization is a preliminary step towards designing a phage for biocontrol.

**Methods:**

In this study, we describe the characterization of a bacteriophage designated phiC119, which can infect and lyse several multidrug-resistant STEC strains and some *Salmonella* strains. The phage genome was screened to detect the *stx*-genes using PCR, morphological analysis, host range was determined, and genome sequencing were carried out, as well as an analysis of the cohesive ends and identification of the type of genetic material through enzymatic digestion of the genome.

**Results:**

Analysis of the bacteriophage particles by transmission electron microscopy showed that it had an icosahedral head and a long tail, characteristic of the family *Siphoviridae*. The phage exhibits broad host range against multidrug-resistant and highly virulent *E. coli* isolates. One-step growth experiments revealed that the phiC119 phage presented a large burst size (210 PFU/cell) and a latent period of 20 min. Based on genomic analysis, the phage contains a linear double-stranded DNA genome with a size of 47,319 bp. The phage encodes 75 putative proteins, but lysogeny and virulence genes were not found in the phiC119 genome.

**Conclusion:**

These results suggest that phage phiC119 may be a good biological control agent. However, further studies are required to ensure its control of STEC and to confirm the safety of phage use.

## Introduction

*Escherichia coli* is an innocuous commensal of the gastrointestinal tract; however, pathogenic *E. coli*, including Shiga toxin-producing *E. coli* (STEC), particularly serotype O157:H7, has been identified as one of the major pathogens causing foodborne diseases (Farfan & Torres, 2012). The Centers for Disease Control and Prevention (Centers for Disease Control Prevention, 2015) estimate that approximately 265,000 illnesses and approximately 4,000 hospitalizations in the United States occur every year due to infections caused by STEC; in developing countries, the situation is often much worse.

Northwestern Mexico is a region that is heavily involved in the production and commercialization of agricultural exports to the US and other countries. Recently, several resistant STEC O157:H7 strains have been isolated from domestic animals on rural farms in this region. The commonality between these strains was multidrug resistance and virulence-encoding genes (Amézquita-López et al., 2012; Canizalez-Roman et al., 2013), which may have potential health risks to humans in the region (Bélanger et al., 2011), as it has been widely documented that several *E. coli* outbreaks worldwide had a zoonotic origin (Jakobsen et al., 2012; Piérard et al., 2012).

Furthermore, antibiotic treatment is contraindicated for STEC infection due to potential worsening of the infection, and alternatives are therefore needed. Implementing strategies to control pathogenic *E. coli* and other foodborne pathogens is a critical step to strengthen food safety in the region. In this regard, among the potential antimicrobial agents, bacteriophages (also called phages) are promising and sustainable agents that can be used against pathogenic bacteria (Mahony et al., 2011; Guenther et al., 2012; Hungaro et al., 2013).

In recent years, interest in the concept of bacteriophages as biocontrol agents has significantly increased. Bacteriophages are viruses that infect bacteria and cause bacterial lysis and are thus considered biocontrol agents for pathogenic bacteria. Desirable candidate phages used for biocontrol should be strictly lytic because they always cause bacterial lysis and release progeny virions (Hagens & Loessner, 2010). Moreover, virulent phages must not integrate their DNA into the host DNA and should display a minimal transduction frequency (negligible rates of transduction); therefore, non-integrating bacteriophages will be the most effective as biocontrol agents. Phages potentially used for biocontrol should be capable of infecting many strains (broad host range) (Chan, Abedon & Loc-Carrillo, 2013; Akhtar, Viazis & Diez-Gonzalez, 2014).

For safety reasons, candidates for biocontrol should not have genes encoding pathogenicity or allergy-triggering proteins. For example, Shiga toxins (*Stx*s) are encoded in the genome of some bacteriophages, and the genetic information encoding *Stx*s can be integrated into the host chromosome (Yan et al., 2011). This type of bacteriophage should be discarded for the purposes of biocontrol because it is possible that the phage could transfer genetic material to the host bacteria. Therefore, a detailed characterization of the bacteriophages is required to provide useful information to determine their potential as biocontrol agents.

Lysogeny-associated, virulence-related and/or antibiotic-resistance genes should be absent in the genome of the bacteriophage, making genome sequencing essential for assessing the safety of a phage (Jun et al., 2015).

Phages have been used by many researchers to biocontrol *E. coli* and other types of bacteria. In all cases, none of the phages reported have been able to lyse all strains. Therefore, it is very important to continue isolating and characterizing novel bacteriophages with broad host ranges against drug-resistant *E. coli* strains prevalent in a given region, which may involve local phage isolation.

In this regard, the new bacteriophage phiC119 isolated in northwestern Mexico (Castro del Campo et al., 2011), exhibited strong in vitro lytic activity against STEC strains, indicating that it could be a candidate biological control agent. However, information on this phage is limited. Therefore, to extend our understanding of the phage characteristics, we describe in this study the characterization of phiC119, providing data that are critical in determining whether it can potentially be used as a biological control agent.

## Materials and Methods

### Bacteriophage, bacterial strain and culture conditions

Bacteriophage phiC119 was previously isolated from horse feces in Sinaloa, Mexico with an enrichment technique. The bacteriophage was isolated from horse feces collected from five different farms located in the region of located in Northwestern Mexico. Briefly, 5 g of horse feces was diluted 1:10 in sterile distilled water (pH 7.0) and gently mixed by inversion. The mixture was cleared by low-speed centrifugation at 6,500 g for 20 min and filtered through a cellulose acetate syringe filter (0.45 μm pore size, GVS filter technology, USA). The 1 mL of filtered supernatant was then mixed with 20 mL exponential phase bacterial culture, and incubated at 37 °C for 18–24 h. After incubation, the bacterial cells were centrifuged and the supernatant was filtered through a 0.22 μm pore size cellulose acetate syringe filter (GVS filter technology, IN, USA). Then, 100 μl of filtrate and 1 mL of the host strain were mixed with soft agar and poured onto an TSA agar plate. After 24 h incubation at 37 °C, plates were checked for a clear zone of bacterial lysis. Single plaques were picked with a sterile glass Pasteur pipette and suspended in 1 mL of sterile distilled water, and each individual plaque was re-isolated three times to ensure the purity of the phage isolate. The phage was stored at −20 °C in tryptic soy broth (TSB, Bioxon, Mexico) containing 30% (v/v) glycerol for further characterization. *E. coli* O157 EC-48 (63-Fv18-1) was previously isolated from fecal samples from domestic animals collected from farms located in the Culiacan Valley and was used as the host for phage propagation in this study. Bacterial strains and phage stocks were obtained from the culture collection maintained by the Food Safety National Research Laboratory (LANIIA) at the Research Center in Food & Development (CIAD), Culiacan station. *E. coli* was grown on TSB at 37 °C; the overnight culture was used in the assays described below.

### Host range

The host range of phage phiC119 was determined with a spotting assay using strains previously described as pathogenic in mammalian cells (Amézquita-López et al., 2014). Additionally, 44 environmental *Salmonella* strains were also included in the study (Jiménez et al., 2014; Estrada-Acosta et al., 2014) (Table 1). On the surface of TSA plates (TSA media with 1.2% agar), 1 mL of overnight culture of each strain and 3 mL of soft agar (TSA media with 0.4% agar) were poured and allowed to solidify. Then, a 10 μL aliquot of several phage dilutions were spotted onto each bacterial overlay and incubated at 37 °C for 18–24 h. After incubation, the presence of phage lysis zones was evaluated in the drops. All testing was performed in triplicate. Bacterial strains used for the bacteriophage host-range investigation were obtained from the LANIIA at the CIAD.

**Table 1:** Bacterial strains used in the host range spectrum of the bacteriophage phiC119. Phage was assessed for host range by spot testing.

Bacterial	Strain	Bacterial lysis
*E. coli* O157:H7	HC14-1	+
*E. coli* O157:H7	HE7-1	+
*E. coli* O157:H7	HC14-2	+
*E. coli* O157:H7	AC6-1	+
*E. coli* O157:H7	HE10-1	−
*E. coli* O157:H7	AR7-2	−
*E. coli* O157:H7	AR17-2	−
*E. coli* O157:H7	AC6-1	−
*E. coli* O157:H7	AR15-1	−
*E. coli* O157:H7	AR17-1	−
*E. coli* O157:H7	RM8744	+
*E. coli* O157:H7	RM8753	+
*E. coli* O157:H7	RM8754	+
*E. coli* O157:H7	RM8759	+
*E. coli* O157:H7	RM8767	+
*E. coli* O157:H7	RM8768	+
*E. coli* O157:H7	RM8769	+
*E. coli* O157:H7	RM8781	+
*E. coli* O157:H7	RM8920	+
*E. coli* O157:H7	RM8921	+
*E. coli* O157:H7	RM8922	+
*E. coli* O157:H7	RM8927	+
*E. coli* O157:H7	RM8928	−
*E. coli* O157:H7	RM9450	+
*E. coli* O157:H7	RM9451	+
*E. coli* O157:H7	RM9452	+
*E. coli* O157:H7	RM9453	+
*E. coli* O157:H7	RM9455	+
*E. coli* O157:H7	RM9457	+
*E. coli* O157:H7	RM9458	+
*E. coli* O157:H7	RM9459	+
*E. coli* O157:H7	RM9462	−
*E. coli* O157:H7	RM9463	+
*Salmonella* Weltevreden	AC2-039	−
*Salmonella* Oranienburg	AC2-041	−
*Salmonella* Saintpaul	AC2-046	−
*Salmonella* Minnesota	AC2-070	+
*Salmonella* Anatum	AC2-079	−
*Salmonella* Oranienburg	AC2-100	−
*Salmonella* Montevideo	CM-02	−
*Salmonella* Saintpaul	AC2-137	−
*Salmonella* Oranienburg	AC2-142	−
*Salmonella* Luciana	AC2-240	+
*Salmonella* Anatum	CM-50	−
*Salmonella* Minnesota	CM-51	−
*Salmonella* Montevideo	CM-52	−
*Salmonella* Agona	AC2-346	−
*Salmonella* Muenster	CM-08	−
*Salmonella* Muenster	AC2-366	−
*Salmonella* Montevideo	AC2-370	−
*Salmonella* Weltevreden	CM-08	−
*Salmonella* Poona	CM-18	−
*Salmonella* Oranienburg	CM-21	−
*Salmonella* Saintpaul	CM-25	−
*Salmonella* Give	CM-31	−
*Salmonella* Saintpaul	AC2-098	−
*Salmonella* Oranienburg	AC2-026	+
*Salmonella* Pomona	AC2-248	−
*Salmonella* Oranienburg	HC2-2	−
*Salmonella* Oranienburg	HC2-1	−
*Salmonella* Oranienburg	HC2-3	−
*Salmonella* Give	HB4-2	−
*Salmonella* Saintpaul	HE4-1	−
*Salmonella* Give	HB4-1	−
*Salmonella* Give	HB4-1	−
*Salmonella* Weltevreden	HD4-2	−
*Salmonella* Give	HB4-3	−
*Salmonella* Saintpaul	HE4-3	−
*Salmonella* Weltevreden	HD4-3	−
*Salmonella* Agona	HD5-1	+
*Salmonella* Give	HD6-3	−
*Salmonella* Oranienburg	HD5-2	−
*Salmonella* Oranienburg	HE6-1	−
*Salmonella* Sandiego	HF6-3	−
*Salmonella* Montevideo	S-188	−
*Salmonella* Oranienburg	S-190	+
*Salmonella* Oranienburg	S-228	−

**Notes:**

+, indicate positive sensitivity to phage lysis.

−, indicate negative sensitivity to phage lysis.

### One-step growth curve

*E. coli* O157 EC-48 was inoculated into 40 mL TSB broth medium and incubated at 37 °C with shaking to reach an OD600 of 0.5. The phage and host cells were mixed with a MOI of 0.01 and allowed to adsorb for 2 min at room temperature. After incubation, the mixture was harvested by centrifugation at 10,000 × g for 1 min at 4 °C. Subsequently, the supernatant was discarded to remove the free phages. The pellet containing infected host cells was gently re-suspended in equal volume of pre-warmed TSB and shake culture at 37 °C. Samples were taken at 5 min intervals (up to 60 min), and phage titer was calculated by double agar plates. The experiment was carried out in triplicated to estimate burst size and latency.

### Bacteriophage propagation and DNA extraction

Bacteriophage propagation was performed using the double-layer plaque technique described by Carey-Smith et al. (2006). Briefly, 100 μL of phage stock was mixed with 1 mL of overnight cultured *E. coli* (CECT 4076) and 2.8 mL of TSB agar (0.4%) preheated to 50 °C. The mixture was poured onto tryptic soy agar (TSA, Bioxon, México) plates (100 × 15 mm Petri dishes) and incubated for 18–24 h at 37 °C under aerobic conditions. Six milliliters of sterile SM buffer (100 mm NaCl, 25 mm Tris-HCl (pH = 7.5), 8 mm MgSO_4_ and 0.01% (w/v) gelatin) was added to the surface of each plate, and the top agar was recovered using a sterile loop. Then, the eluate was centrifuged at 4,500 × g for 10 min at 4 °C, and the supernatant was recovered; the procedure was repeated twice. The final pooled supernatant was filtered through a cellulose acetate syringe filter with a 0.45 μm pore size (GVS filter technology, IN, USA). The phage filtrate was concentrated by centrifugation at 40,000 × g for 2 h, and then the pellet was gently resuspended by pipetting in 10 mL of SM buffer and filtered using a cellulose acetate syringe filter with a 0.20 μm pore size. The bacteriophage titer was determined by a double-layer plaque technique with serial decimal dilutions of phage concentrate. The final purified phages were stored at 4 °C.

One milliliter of purified phage suspension (approximately 1 × 10^12^ plaque forming units (PFU) per mL) was incubated with 10 μL of DNase I/RNase A (10 mg/mL) (Sigma-Aldrich, MO, USA) for 1 h at 37 °C. Phage DNA was extracted using SDS-proteinase K method as previously described (Sambrook & Russell, 2001). Phage DNA was stored at 4 °C until use. The nucleic acid extract was subjected to digestion with DNase I and RNase according to the manufacturer’s instructions.

### Transmission electron microscopy and plaque characteristics

Thirty microliters of purified phage suspension was adsorbed to carbon-coated copper grids (400-mesh) in a vacuum evaporator (JEE400, JEOL Ltd. Tokyo, Japan), allowed to air dry and then negatively stained with 2% phosphotungstic acid (pH 7.2). The excess solution was absorbed with filter paper, and samples were observed with a transmission electron microscope (JEM-1011, JEOL Ltd. Tokyo, Japan) operating at 80 kV (López-Cuevas et al., 2011).

Bacteriophage plaques formed on a TSA plate during the process of propagation (using dilutions that generated 15–30 plaques per plate) were analyzed according to the procedure described by Gallet, Kannoly & Wang (2011) with minor modifications. Briefly, images of ten plates were captured by a supersensitive high-resolution 16-bit camera that was deeply cooled for faint image detection (Bio-Rad Laboratories), and the image of five plaques for each plate were displayed with the ImageJ software (developed at the National Institutes of Health, Bethesda, Maryland). The plates were then incubated for 18–24 h at 37 °C before plaque size determination. To calculate the surface area (expressed in square millimeters) corresponding to each pixel, a graticule of 1 mm^2^ was used as the reference scale for the simplified measurement of the lysis plaques. According to the analysis, each pixel corresponded to 0.5 mm^2^.

### PCR to identify *stx_1_* and *stx_2_* encoding bacteriophage

Multiplex PCR using a GoTaq® PCR Core System I (Promega, WI, USA) was performed to determine the presence of the *stx_1_* and *stx_2_* genes in the genome of phage phiC119. PCR assays were performed using the protocol previously described by Paton & Paton (1998). In addition, *E. coli* O157:H7 (CECT 4076) DNA was included in the PCR screen as a positive control. All primers used in the PCR assays were commercially synthesized by Sigma–Aldrich (Toluca, México).

### Genome size estimation and analysis of the cohesive ends

The genome ends were determined as described by Casjens & Gilcrease (2009). Briefly, 1 μg of phage genetic material was digested with the restriction enzyme *Eco*RV according to the manufacturer’s specifications, followed by heating for 15 min at 75 °C. Subsequently, the reaction mixture was divided into two equal parts. One was rapidly cooled by immersion into an ice-water bath for 10 min, and the other was cooled to room temperature prior to electrophoresis on a 1% agarose gel at a voltage of 75 V for 90 min. They were then stained with ethidium bromide (1 μL mL^−1^), and images were captured using a ChemiDoc™ MP imaging system with Image Lab™ software (Bio-Rad Laboratories). The lambda phage DNA was used as a positive control. Lambda DNA digested with the *Hind*III endonuclease was used as a standard molecular weight marker (Promega, WI, USA).

### Genome sequencing and annotation

DNA sequencing was performed at the National Laboratory of Genomics for Biodiversity (LANGEBIO) using the MiSeq sequencing system (Illumina, Inc.) (150-bp single-end reads). In total, 4,832,127 reads were generated and assembled into one contig using Geneious v8.1.2 (the final sequence coverage was approximately 50×). The sequence assembly was validated by a comparative restriction profile (Promega, WI, USA). Potential open reading frames (ORFs) longer than 100 bp were predicted by GeneMark (http://exon.gatech.edu/) and ORF Finder (http://www.ncbi.nlm.nih.gov/gorf/gorf.html). The putative ORFs were analyzed by BLAST at the National Center for Biotechnology Information (NCBI) (http://blast.ncbi.nlm.nih.gov/Blast.cgi) against the database of non-redundant protein sequences using a significant *E*-value of 10^−3^. Moreover, all identified ORFs were compared against the virulence factor database (http://www.mgc.ac.cn/VFs/) (Chen et al., 2012) and the ResFinder database (http://cge.cbs.dtu.dk/services/ResFinder/) (Kleinheinz, Joensen & Larsen, 2014). The predicted phage protein sequences were searched to identify proteins that were potentially allergenic using tools available at http://www.allergenonline.com from the Food Allergy Research. This analysis was complemented with a search for conserved protein domains using InterProScan, HMMER, Prosite, Motif Search and SMART. Hypothetical isoelectric points and the molecular weights of putative proteins were predicted using the ExPASy server (http://us.expasy.org/tools/protparam.html). Potential tRNA genes in the genome sequence were predicted using tRNAscan-SE and ARAGORN. Promoters and potential rho-independent terminators were identified using the Neural Network Promoter Prediction tool of the Berkeley Drosophila Genome Project (http://www.fruitfly.org/seq_tools/promoter.html) and the FindTerm program (http://linux1.softberry.com/berry.phtml?topic=findterm&group=programs&subgroup=gfinb)(energy threshold value: −11), respectively. The nucleotide genome sequence of phage phiC119 has been deposited in the GenBank database under accession number KT825490.

The lifestyle of the phages was predicted using the PHACTS program (http://www.phantome.org/PHACTS/upload.php). Statistical analysis was performed using Minitab statistical software version 14 (Minitab Inc., State College, PA, USA). Hierarchical clustering analysis was used to determine the relationship between genome size, gene density, and lifestyle.

Furthermore, the amino acid sequences of terminase large subunits of phiC119 and others phages were obtained from GenBank. Twelve bacteriophages, including the phiC119, were selected for phylogenetic analysis, these phages were selected as being the most well-known representatives of each important family of phages. The amino-acid-sequences were aligned using the program ClustalW, and the neighbor-joining phylogenetic tree was generated using Geneious v8.1.2.

## Results

### Bacteriophage, bacterial strain and culture conditions

**Figure 1:**
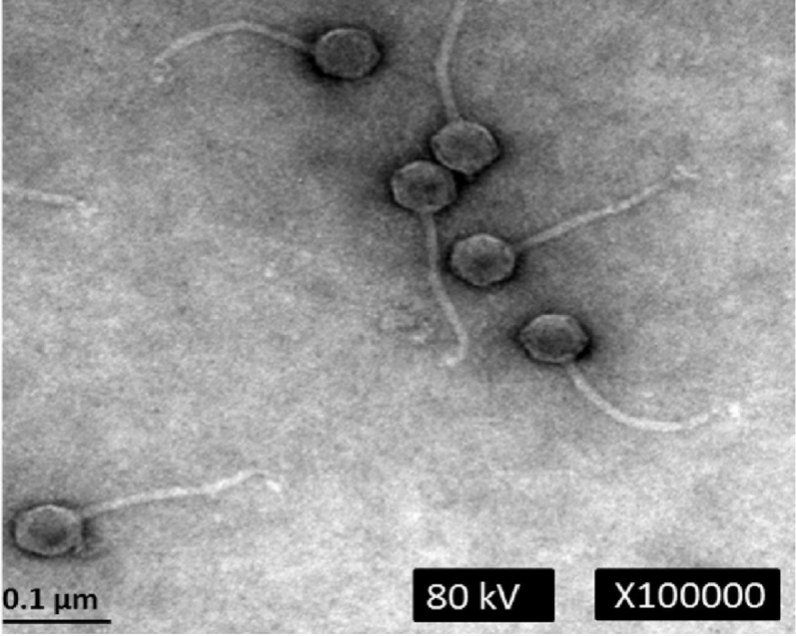
Transmission electron micrograph of phage phiC119 negatively stained with 2% unanyl acetate. Phage phiC119 showing typical *Siphoviridae* morphology, which exhibit a noncontractile tail with a length of 168–172 nm. The icosahedral head of phiC119 has a length of 43–45 nm and a width of 7–9 nm. The bar indicates 100 nm.

Electron microscopic analysis revealed that phage phiC119 was non-enveloped with an icosahedral capsid of approximately 43–45 nm in diameter and a tail of 168–172 nm in length and 7–9 nm in width. These characteristics suggest that phage phiC119 is a member of the *Siphoviridae* family. The flexibility and the uniformity of the tail lengths indicated that it was non-contractile (Fig. 1). Phage phiC119 produced very large (1.0–1.5 mm in diameter), clear and uniform-sized plaques after 18–24 h incubation at 37 °C with *E. coli* O157:H7 EC-48 (63-104 Fv18-1) using the double-agar overlay technique.

### Host range

The bacteriophage phiC119 was recently isolated by our lab from horse feces and to determine the susceptibility of bacterial strains to lysis by phage, thirty-three environmental isolates of *E. coli*, previously isolated at the CIAD, were used for determine the host range of phage phiC119 (Table 1). A high proportion (75.75%, n = 25) of *E. coli* strains were sensitive to phage phiC119, which formed plaqueson a broad spectrum of *E. coli* serogroups O157, including *Stx*-producing *E. coli*. These *E. coli* isolates were previously characterized as highly virulent because they exhibit toxicity against mammalian cells and have high levels of antibiotic resistance (Amézquita-López et al., 2014; Amézquita-López et al., 2016).

Additionally, we determined the host range of the phage phiC119 with a collection of 44 *Salmonella* strains. Interestingly, the phage was also able to infect only some strains of certain *Salmonella* serotypes (Oranienburg, Agona, Luciana, and Minnesota). However, the phage was not able to lyse the other bacterial species used in this study.

### One-step growth curve

**Figure 2:**
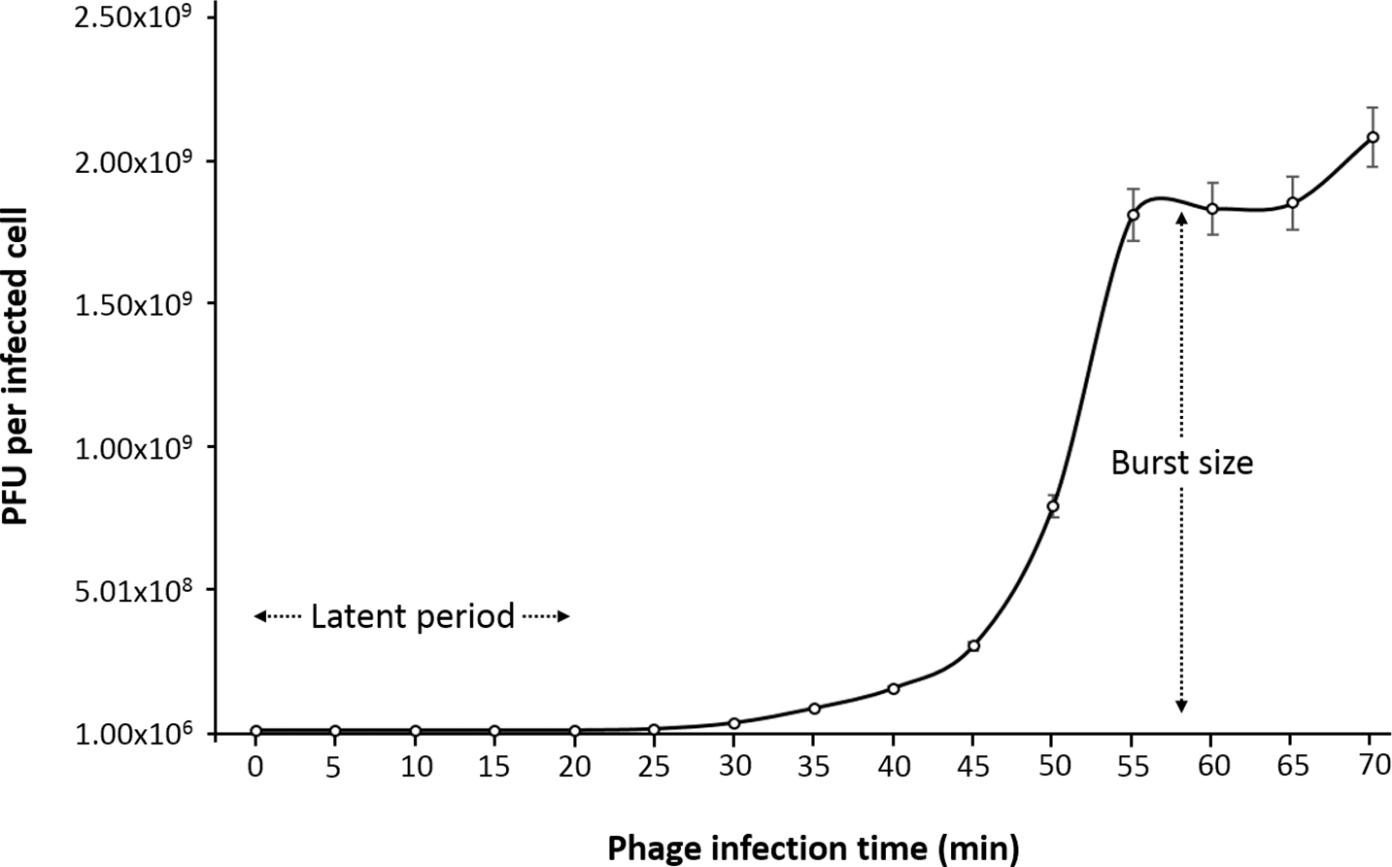
One-step growth curve of phage phiC119 on *E. coli* at 37 °C. The parameters of phage growth are indicated in the figure, showing the latent period (20 min) and the average burst size (210 viral particles per host cell). Means ± standard error from three independent experiments are shown. Some of the error bars were too small to be visible.

One-step growth curve experiment was performed to determine the latent time period and burst size of the phage, as these are two of the most important characteristics of phage infection process. According to the results obtained, the entire phiC119 life cycle takes about 60 min to complete. phiC119 had approximately 20 min of latent period and the average burst size is 210 phage particles per infected cell after 55 min at 37 °C (Fig. 2).

### Detection of the *stx* genes

**Figure 3:**
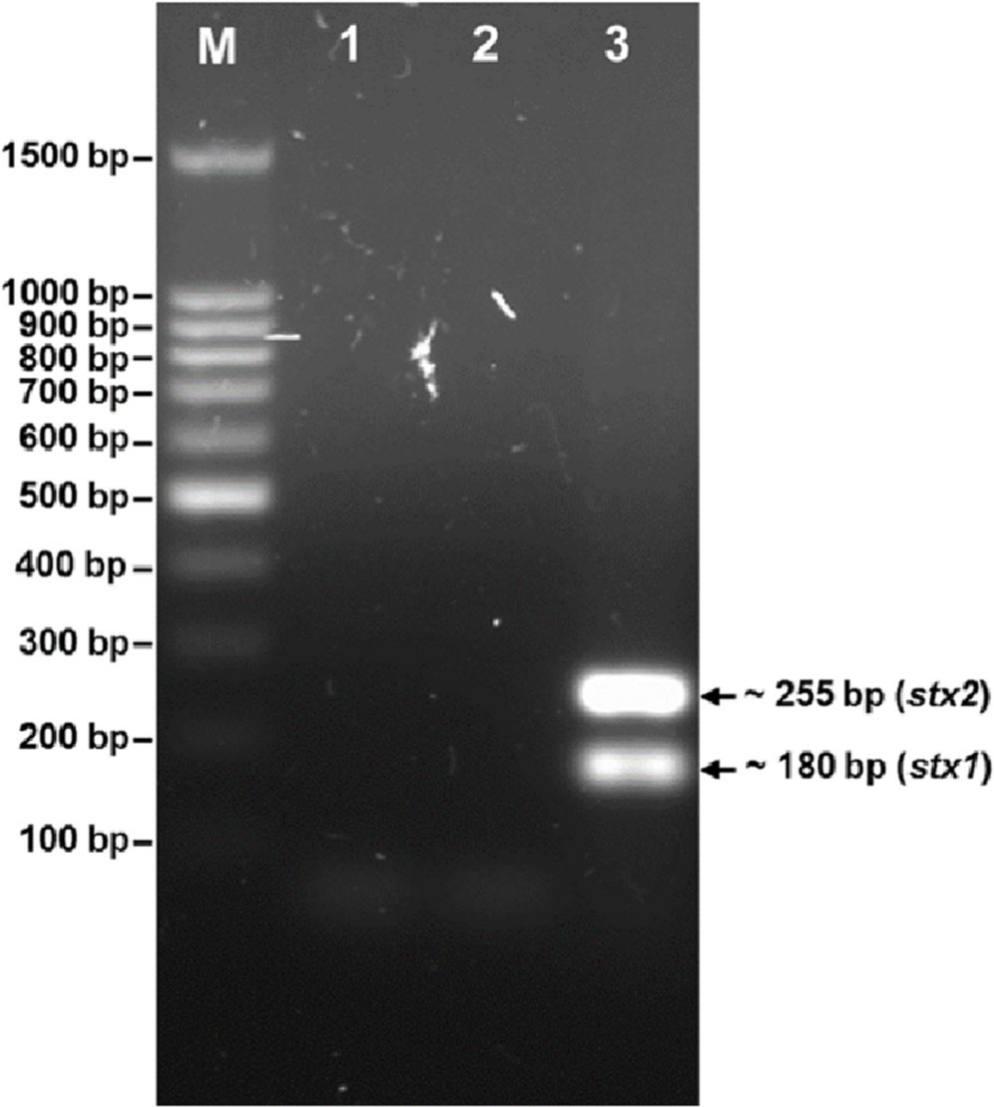
Agarose gel electrophoresis of PCR products amplified from DNA extracted from phage phiC119. PCR was performed to detect the presence of *stx_1_* and *stx_2_* genes in phage genome. The size of *stx_1_* and *stx_2_* amplicon corresponds to the 180 and 255 bp band, respectively. Lane M; 100 bp DNA ladder (Promega), Lane 1; negative control, Lane 2; Bacteriophage phiC119 sample, Lane 3; positive control.

The phage was tested for the presence of the *stx_1_* and *stx_2_* genes (Fig. 3). PCR screening for the *stx* genes using DNA isolated from bacteriophage phiC119 was negative. However, other virulence factors may be encoded in the bacteriophage genome, and therefore, genome sequencing and in silico analyses are required to ensure the absence of virulence, antibiotic resistance or lysogenic genes because lysogenic conversion can increase the pathogenic potential of the bacteria towards their hosts. Hence, bacteriophages suitable for biocontrol purposes should not encode virulence genes or potential immunoreactive allergens.

### Analysis of the cohesive ends

**Figure 4:**
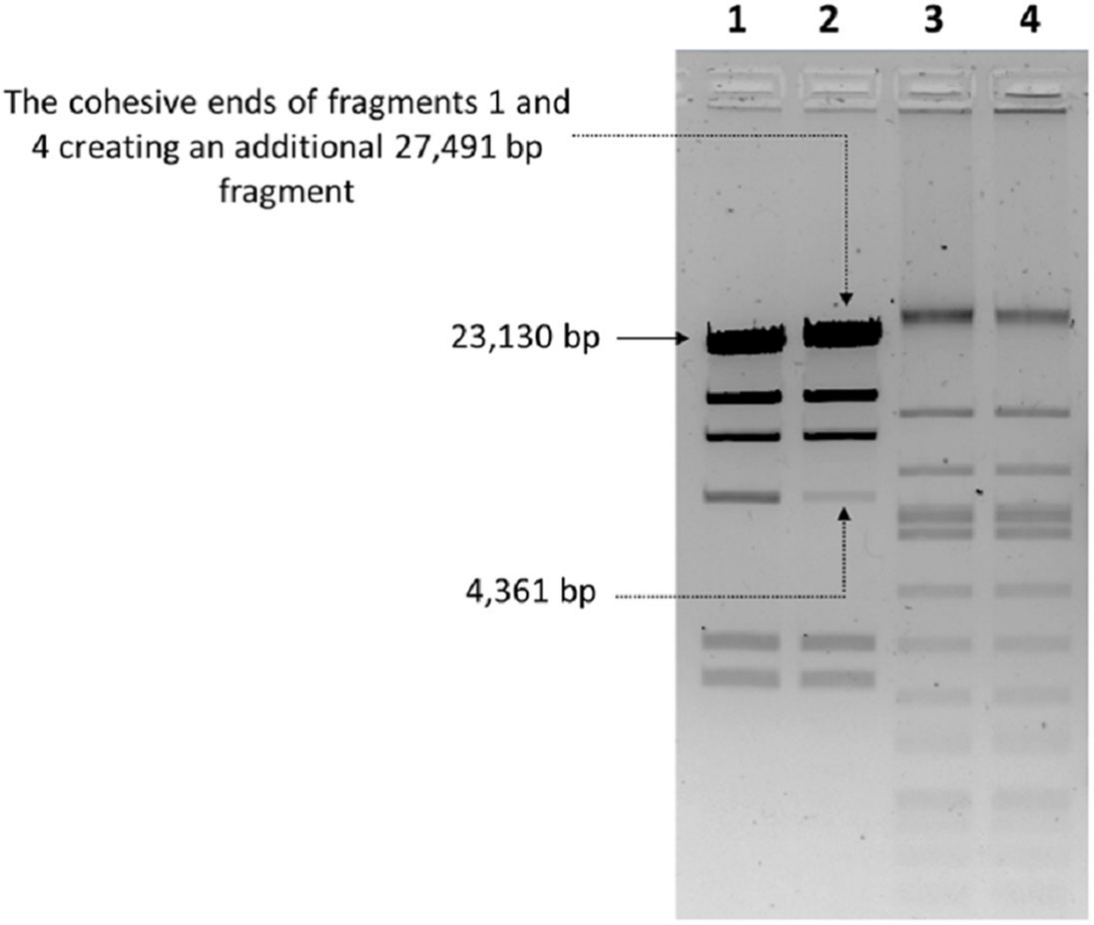
Endonuclease digestion analysis of phage phiC119 genomic DNA. Phage genomic DNA was digested with the restriction enzyme *Eco*RV. The digested DNA fragments were separated by 1% agarose gel electrophoresis. *Hind*III-digested lambda DNA was used as a positive control to detect annealing of cohesive ends (Lane 1 and 2) and phiC119 DNA digested with *Eco*RV (Lane 3 and 4). After digestion, lines 1 and 3 were rapid cooling by immersion into an ice-water bath for 10 min, and the lines 2 and 4 cooled to room temperature. The arrows indicate fragments that bind to be cohesive in positive control.

**Figure 5:**
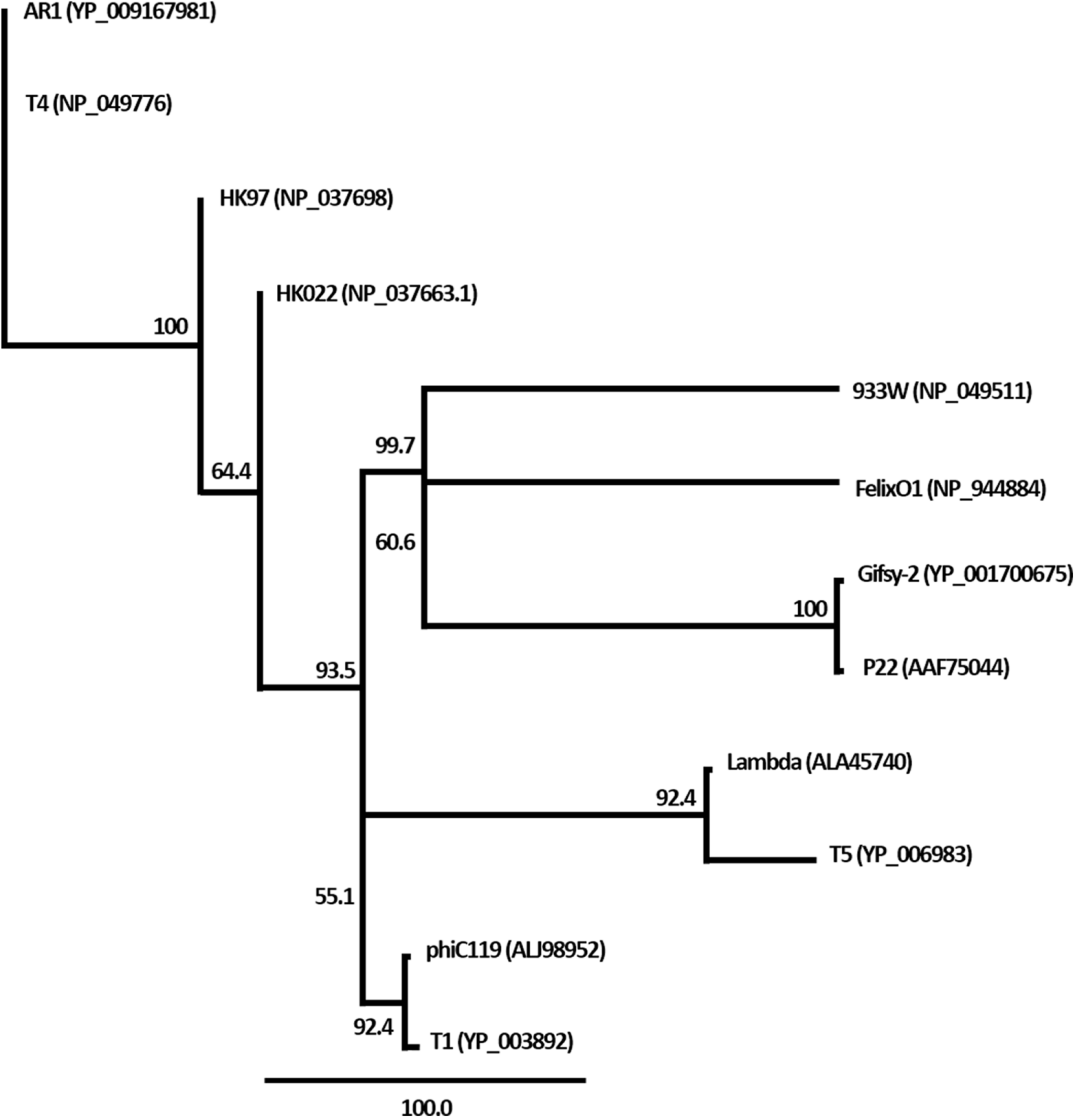
Phylogenetic analysis of the terminase large subunits of phage phiC119 and other large terminase genes from diverse phage genomes. Numbers on the branches are bootstrap values.

The nucleic acid of phage phiC119 was resistant to RNase, sensitive to DNase and digested by restriction enzymes. These results indicate that the phage genome is double-stranded DNA and is approximately 47 kb in size (genome size estimated from the digested fragments). Moreover, enzymatic digestion of the genome suggested that phage phiC119 utilizes the *pac*-mechanism of DNA packaging because heating/cooling of DNA after enzymatic digestion did not alter the restriction patterns (Casjens & Gilcrease, 2009) (Fig. 4). There, was no evidence for the existence of cohesive ends in the bacteriophage genome. In addition, the analysis revealed a close a phylogenetic relationship between the phagephiC119 and other *pac*-type phages (Fig. 5).

### Bacteriophage genome features

**Figure 6:**
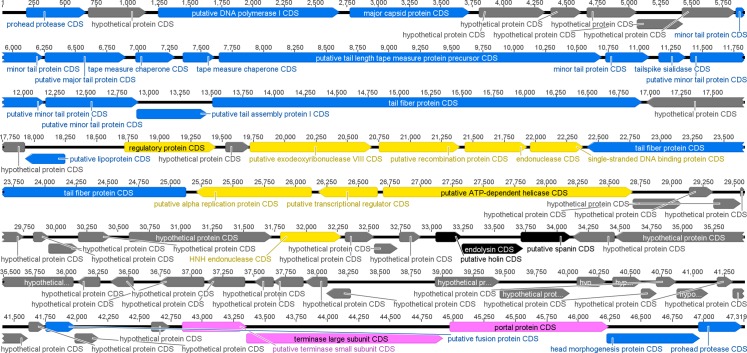
Graphic representation of genome organization of the phage phiC119. Putative ORFs are indicated as arrows, the orientation of which shows the direction of transcription. The colors were assigned according to the possible function of each ORF. Morphogenesis (blue), DNA replication (yellow), lisis (black), DNA packaging (pink), and hypothetical genes with unknown function (gray).

Overall, the bacteriophage genome contained 75 putative ORFs (90.4% of the genome consists of a coding region) (Fig. 6), 21 of which are transcribed from the complementary strand. Based on sequence similarities and protein domains/motifs and BLAST searches, 42 genes were assigned to conserved sequences and 33 were sorted into known functional categories. Furthermore, bioinformatics analysis revealed an organization of the phage genome into four functional modules, coding for structural proteins, DNA packaging, replication and host lysis (a detailed description of gene functions is shown in Table S1).

The genome sequence of phiC119 consisted of 47,319 bp with an average GC content of 44.20%, which is significantly lower than that of *E. coli* (average 50%). Furthermore, a tRNA gene was identified (Arg-tRNA (anti-codon CCT)) between positions 42,465–42,540 in an adjacent region to the morphogenetic cluster, indicating probable involvement in phage morphogenesis.

Bacteriophage phiC119 genome possesses a high gene density (1.60 genes per kilobase), it contains a large proportion of genes that overlap with coding regions of neighboring genes. Similarly, different authors have indicated that the genes of coliphages (bacteriophages that infect *E. coli* hosts) are usually tightly packed together with small intergenic regions and a high gene density (Miller et al., 2003; Santos & Bicalho, 2011). Moreover, the genome of phage phiC119 contains several overlapping sets of genes; 20 ORFs overlap with an adjacent ORF, thus generating an increase in the density of genetic information.

Genomic analysis showed that phage phiC119 does not have lysogenic genes, such as integrase and repressor genes. In addition, lifestyle prediction using the PHACT program suggested that phiC119 is a virulent bacteriophage. Furthermore, the bioinformatics analysis of the phiC119 phage did not find any undesired genes in its genome, indicating the lack of known genes coding for potential allergens and virulence genes. Therefore, bacteriophage phiC119 has two of the desirable features of candidate phages used for biocontrol.

#### Morphology module

Genomic analyses revealed that at least 18 ORFs are involved in the morphogenesis of bacteriophage phiC119. The products of putative ORFs 1 and 75 shared identity with prohead proteases, suggesting that these ORFs are necessary for capsid morphogenesis. Moreover, phage phiC119 possesses a potential major capsid protein encoded by ORF 4. The tail proteins were identified as ORFs 10, 11, 14, 15, 17, 18, and 19. Additionally, ORFs encoding tailspike and two tail fiber proteins were found. According to Yamashita et al. (2011), these structures are required for specific recognition and binding to the host receptor and were identified as ORF 16 and ORF 20 and 29, respectively. Phage phiC119 encodes two tape measure chaperone proteins (ORFs 12 and 13).

#### Nucleotide metabolism module

We also identified ORFs involved in nucleotide metabolism including ORF 3, which encodes DNA polymerase I, an enzyme used during DNA replication of the bacteriophage. The product of ORF 23 encodes a regulatory protein, which is an essential enzyme for DNA transcription.

ORFs 25, 26 and 27 encode, respectively, exodeoxyribonuclease VIII, a recombination protein and an endonuclease. Assays performed by Pickard et al. (2008) have shown that these proteins are essential for proper DNA packaging, and therefore, these proteins may have comparable roles in phage phiC119.

The putative ORF 28 encodes a protein with conserved motifs associated with a single-stranded DNA binding protein. Single-stranded DNA-binding proteins promote the integration of components of the DNA replication complex (Hollis et al., 2001). This protein is likely essential for DNA replication of phage phiC119. Phage phiC119 contains an alpha replication protein, a putative transcriptional regulator and an ATP-dependent helicase (ORFs 30, 31 and 32), which are all proteins involved in DNA replication (Hua et al., 2014). ORF 41 is closely related to an HNH endonuclease that participates in phage DNA repair (Moodley, Maxwell & Kanelis, 2012).

#### Lysis module

A total of three putative ORFs encoding proteins associated with the lysis of the host were found; we determined that ORF 45 encodes holin, a protein that permeabilizes the inner membrane, oligomerizes in the host cell membrane and forms large pores that are utilized as transport channels for endolysin to access and degrade the peptidoglycan layer (Shin et al., 2014). Moreover, the lysis module includes ORF 46, which encodes a protein sharing 84% identity with an endolysin. Analysis of ORF 46 revealed one conserved motif with lysozyme. The presence of this motif suggests that this protein is probably an enzyme involved in peptidoglycan cleavage (Xu et al., 2015). The product of ORF 47 shared over 94% identity with a spanin, a small lipoprotein that is required for disruption of the outer membrane (Berry et al., 2012).

## Discussion

Phages have been used by many researchers to biocontrol *E. coli* and others types of bacteria. In all cases, none of the phages reported have been able to lyse all strains. The present study describes a new bacteriophage, designated phiC119, including a description of its morphology, host range, analysis of the cohesive ends and genome sequence.

Transmission electron microscopy revealed that the bacteriophage phiC119 belongs to the order *Caudovirales* as a member of the *Siphoviridae* family according to classifications proposed by the International Committee on Taxonomy of Virus. These results are consistent with previous reports on bacteriophages because approximately 95% of phage isolates are classified in the order *Caudovirales* (Swanson et al., 2012). Furthermore, within approximately 4 h, phage phiC119 formed large and clear plaques, which is associated with phages that possess a lytic cycle (Kwiatek et al., 2015). Previous research suggested that bacteriophages that produce larger plaques generally have a larger burst size, indicative of lytic phages (Abedon & Culler, 2007).

Phage phiC119 has strong lytic activity against the *E. coli* strains used in this study. Many of the *E. coli* strains are multidrug resistant and pathogenic in mammalian cells (Amézquita-López et al., 2012; Amézquita-López et al., 2014). Moreover, the phage was able to lyse some strains of *Salmonella* serotypes such as Minnesota, Luciana, Oranienburg, and Agona, suggesting that phage phiC119 can be considered a broad host range phage and may be an effective biocontrol agent, as phages with broad host range activity against STEC strains are advantageous in biocontrol (Niu et al., 2012). The potential for lysis of the highest number of strains is important for the potential use of bacteriophages in biocontrol of the bacterial pathogens (Eyer et al., 2007). Therefore, based on broad host range against STEC strain, we suggest that phiC119 should be considered a good candidate for biocontrol.

Biological characterization of the phage revealed that phiC119 has an average burst size of 210 PFU per infected cell with an average latent period of 20 min, indicating that phiC119 has strong lysis. Phages with high burst sizes are more effective to biocontrol and phage therapy (Abedon, Herschler & Stopar, 2001). According to the one-step growth curve results, phiC119 can be considered as a candidate for biocontrol evaluation.

Genetic analyses suggest that the bacteriophage genome is organized into functional modules. This modular organization allows genes that are involved in the same biological process to be clustered in the same module, which is common in most tailed bacteriophages (Haddad et al., 2014; Teng et al., 2015). Furthermore, the phage does not have cohesive ends. In this regard, Casjens & Gilcrease (2009) argued that phages with the *pac*-mechanism (called headful packaging) are able to produce transduction. However, most new viral particles generated in such process are expected to be nonviable with defective replication functions and are eliminated by natural selection (Krupovic et al., 2011). In contrast, recent reports suggested that *cos*-type phages represent a novel mechanism of horizontal gene transfer, although at a lower frequency than *pac*-type phages.

The restriction profiles indicated the absence of cohesive ends in phiC119 phage genome. To determine the most probable packaging strategy used by this phage, phylogenetic tree was constructed by comparing the amino acid sequences of terminase proteins of the most well-known representatives of each important family of phages, including the phiC119.

The terminase in phage phiC119 showed 62.1% sequence identity with that of Enterobacteriophage T1, this phage packages its DNA via a headful packaging mechanism (Roberts, Martin & Kropinski, 2004). Considering that terminase determines the DNA-packaging strategy of the phage (Casjens & Gilcrease, 2009), phylogenetic analysis suggests that the phage phiC119 packages DNA by a headful mechanism similar to that of T1. This is in agreement with the restriction endonuclesae digestion analysis.

The genome sequence of phiC119 consisted of 47,319 bp with a GC content of 44.20%, a value lower than that of its hosts. This observation is consistent with previous reports showing that virulent phages are on average 4% poorer in GC content than their hosts, while in temperate bacteriophages, the guanine content is usually very close to the host (Rocha & Danchin, 2002). The low GC content of phage genome suggests that phage phiC119 might have acquired the ability to infect *E. coli* strains over a long period of time (Kwan et al., 2006; Jin et al., 2014). Additionally, genome size is an important biological property of the virus, as the genome size determines the numbers of proteins encoded by the phage and is correlated with virion complexity, although there are some exceptions (Abedon, 2011). These results suggest that phiC119 is a bacteriophage with low structural complexity; this is consistent with transmission electron microscopic observations of the phiC119 bacteriophage.

Phage phiC119 has overlapping ORFs, overlapping genes is a common phenomenon in phage genomes, which is a tactic to minimize genome size. Thus, this represents the compression of a large amount of genetic information into short nucleotide sequences without a loss of protein function (Pavesi, 2006). This strategy also plays a fundamental role in transcriptional and translational regulation of gene expression (Johnson & Chisholm, 2004).

It is possible that phage phiC119 expresses structural proteins in a more efficient way because phages encoding tRNAs can overcome possible differences in codon usage between the phage and the host (Samson & Moineau, 2010). The presence of tRNAs is common in strictly virulent or lytic phages (Santos et al., 2011). From a biological point of view, the existence of tRNAs in the phage genome suggests that phiC119 may have a short latent period and a large burst size because a previous study revealed that tRNAs enable phages to improve propagation and increase the kinetics of viral replication, as tRNAs are related to optimal codon usage (Jun et al., 2014).

Comparative analysis of genes at the amino acid sequences using the BLASTP program revealed that the tail fiber proteins of phiC119 (protein_id = ALJ98900.1 and ALJ98909.1) are homologous to tail fiber proteins of phages that infect the members of the bacterial family *Enterobacteriaceae*, including phage that infect *Salmonella* and *E. coli*. Phage specificity is largely determined by the tail fiber’s ability to bind to specific structures on the surface of bacteria. The similarities of the tail fiber proteins could imply that these phages in general have the same host range (Haggård-Ljungquist, Halling & Calendar, 1992). This may be the main reason for the polyvalent activity on *Salmonella* and *E. coli* O157:H7 by phiC119.

Analysis of the genome sequence of bacteriophages considered for use as a biocontrol agent is essential. This is to ensure that the phage is strictly lytic and does not encode any phage lysogeny factors, virulence-related genes and/or antibiotic resistance genes (Endersen et al., 2015). The complete genomic sequence analysis of bacteriophage phiC119 revealed the absence of virulence-encoding genes, potential immunoreactive allergens, and lysogeny genes.

In conclusion, transmission electron microscopy revealed that phage phiC119 belongs to the *Siphoviridae* family. Furthermore, phage phiC119 exhibited a broad host range. Genomic analysis suggests that phage phiC119 does not establish a lysogenic state and has no known toxic genes, potential allergens or integrases. These results indicate that phage phiC119 exhibits a number of properties suitable for application as a biocontrol agent for STEC strains. However, further toxicity studies are required to ensure the safety of the phage. Therefore, our future research will be aimed at characterizing this phage for a better understanding of its potential as a biocontrol agent.

## Supplemental Information

10.7717/peerj.2423/supp-1 Supplemental Information 1 Table S1. Features of the open reading frames of bacteriophage phiC119. Functional annotation results were obtained by homology in the GenBank database using BLAST.

## Additional Information and Declarations

### DNA Deposition

The following information was supplied regarding the deposition of DNA sequences: GenBank: accession number KT825490.

### Data Deposition

The following information was supplied regarding data availability: The raw data has been supplied as Supplemental Dataset Files.
