# Supplementary material for: Characterization of novel bacteriophage phiC119 capable of lysing multidrug-resistant Shiga toxin-producing Escherichia coli O157:H7
Source: PeerJ. 2016 Sep 13;4:e2423. doi: 10.7717/peerj.2423 (PMC5028729; doi:10.7717/peerj.2423)
Supplement: Supplemental Information 1 — Functional annotation results were obtained by homology in the GenBank database using BLAST. [file peerj-04-2423-s001.docx]

**Supplementary Table 1.** Features of the open reading frames of bacteriophage phiC119. Functional annotation results were obtained by homology in the GenBank database using BLAST.

| ORF | Start | Stop | Strand | Homology | Query cover | E-value | Ident |
| --- | --- | --- | --- | --- | --- | --- | --- |
| 1 | 191 | 673 | + | Prohead protease [Escherichia phage bV_EcoS_AHS24] | 97% | 2e-88 | 89% |
| 2 | 685 | 1152 | + | Hypothetical protein Rogue1_0020 [Enterobacteria phage vB_EcoS_Rogue1] | 99% | 1e-95 | 92% |
| 3 | 1246 | 2691 | + | Hypothetical DNA polymerase I [Escherichia phage Jk06] | 50% | 3e-62 | 72% |
| 4 | 2771 | 3730 | + | Major capsid protein [Enterobacteria phage vB_EcoS_Rogue1] | 98% | 0.0 | 99% |
| 5 | 3801 | 4331 | + | Hypothetical protein [Escherichia phage EB49] | 97% | 3e-57 | 53% |
| 6 | 4374 | 4619 | + | Hypothetical protein Rogue1_0024 [Enterobacteria phage vB_EcoS_Rogue1] | 98% | 1e-37 | 100% |
| 7 | 4664 | 5065 | + | Hypothetical protein Rogue1_0025 [Enterobacteria phage vB_EcoS_Rogue1] | 99% | 1e-79 | 97% |
| 8 | 5062 | 5430 | + | Hypothetical protein [Escherichia phage e4/1c] | 99% | 9e-80 | 98% |
| 9 | 5423 | 5851 | + | Phage-related hypothetical protein [Escherichia phage Jk06] | 99% | 2e-93 | 96% |
| 10 | 5853 | 6239 | + | Minor tail protein [Escherichia phage bV_EcoS_AHS24] | 99% | 1e-85 | 97% |
| 11 | 6254 | 6904 | + | Putative major tail protein [Enterobacteria phage vB_EcoS_Rogue1] | 99% | 4e-155 | 99% |
| 12 | 6978 | 7292 | + | Tape measure chaperone protein [Citrobacter phage Stevie] | 98% | 2e-26 | 55% |
| 13 | 7355 | 7609 | + | Tape measure chaperone [Citrobacter phage Stevie] | 98% | 3e-24 | 55% |
| 14 | 7644 | 10697 | + | Putative tail length tape measure protein precursor [Escherichia phage e4/1c] | 99% | 0.0 | 99% |
| 15 | 10729 | 11079 | + | Minor tail protein [Escherichia phage bV_EcoS_AHP42] | 99% | 1e-78 | 98% |
| 16 | 11152 | 11367 | + | Tailspike sialidase [Enterobacterio phage phiKP26] | 97% | 8e-43 | 100% |
| 17 | 11401 | 12165 | + | Putative minor tail protein [Enterobacteria phage vB_EcoS_Rogue1] | 99% | 0.0 | 99% |
| 18 | 12175 | 12921 | + | Putative minor tail protein [Enterobacteria phage vB_EcoS_Rogue1] | 99% | 0.0 | 99% |
| 19 | 12902 | 13468 | + | Putative tail assembly protein I [Escherichia phage e4/1c] | 99% | 1e-96 | 98% |
| 20 | 13510 | 16935 | + | Tail fiber protein [Escherichia phage bV_EcoS_AHP24] | 98% | 0.0 | 99% |
| 21 | 16966 | 17928 | - | Hypothetical protein AHP42_26 [Escherichia phage bV_EcoS_AHP42] | 99% | 0.0 | 99% |
| 22 | 17928 | 18248 | - | Putative lipoprotein [Escherichia phage bV_EcoS_AHP42] | 75% | 1e-43 | 99% |
| 23 | 18720 | 19451 | - | Regulatory protein [Escherichia phage bV_EcoS_AKS96] | 93% | 3e-121 | 76% |
| 24 | 19525 | 19719 | - | Hypothetical protein Rogue1_0042 [Enterobacteria phage vB_EcoS_Rogue1] | 98% | 1e-38 | 98% |
| 25 | 19723 | 20691 | - | Putative exodeoxyribonuclease VIII [Enterobacteria phage vB_EcoS_Rogue1] | 99% | 0.0 | 99% |
| 26 | 20748 | 21395 | - | Putative recombination protein [Enterobacteria phage vB_EcoS_Rogue1] | 99% | 1e-141 | 99% |
| 27 | 21437 | 21931 | - | Endonuclease [Salmonella phage FSL SP-126] | 96% | 4e-42 | 48% |
| 28 | 21955 | 22377 | - | Single-stranded DNA binding protein Ssb [Escherichia phage bV_EcoS_AHP42] | 99% | 1e-78 | 98% |
| 29 | 22409 | 25126 | - | Tail fiber [Escherichia phage bV_EcoS_AHP42] | 99% | 0.0 | 99% |
| 30 | 25205 | 26128 | - | Hypothetical alpha replication protein [Escherichia phage Jk06] | 99% | 0.0 | 99% |
| 31 | 26181 | 26654 | - | Putative transcriptional regulator [Enterobacteria phage vB_EcoS_Rogue1] | 91% | 1e-101 | 100% |
| 32 | 26696 | 28690 | + | Putative ATP-dependent helicase [Enterobacteria phage vB_EcoS_Rogue1] | 97% | 0.0 | 99% |
| 33 | 28690 | 29079 | + | Hypothetical protein Rogue1_0050 [Enterobacteria phage vB_EcoS_Rogue1] | 99% | 7e-89 | 100% |
| 34 | 29138 | 29332 | + | Hypothetical protein [Escherichia phage e4/1c] | 98% | 3e-36 | 100% |
| 35 | 29332 | 29550 | + | Hypothetical protein Rogue1_0052 [Enterobacteria phage vB_EcoS_Rogue1] | 98% | 3e-44 | 100% |
| 36 | 29547 | 29747 | + | Hypothetical protein Rogue1_0053 [Enterobacteria phage vB_EcoS_Rogue1] | 98% | 3e-41 | 100% |
| 37 | 29822 | 29947 | + | Hypothetical protein Rogue1_0054 [Enterobacteria phage vB_EcoS_Rogue1] | 97% | 2e-19 | 95% |
| 38 | 29944 | 30183 | + | Hypothetical protein Rogue1_0055 [Enterobacteria phage vB_EcoS_Rogue1] | 86% | 6e-44 | 100% |
| 39 | 30180 | 30437 | + | Hypothetical protein [Escherichia phage e4/1c] | 98% | 3e-54 | 100% |
| 40 | 30589 | 31725 | + | Hypothetical protein AHP42_45 [Escherichia phage bV_EcoS_AHP42] | 99% | 0.0 | 99% |
| 41 | 31791 | 32282 | + | HNH endonuclease [Escherichia phage bV_EcoS_AKS96] | 99% | 1e-102 | 98% |
| 42 | 32311 | 32541 | + | Hypothetical protein [Escherichia phage e4/1c] | 98% | 8e-45 | 99% |
| 43 | 32541 | 32729 | + | Hypothetical protein [Escherichia phage e4/1c] | 98% | 2e-37 | 100% |
| 44 | 32742 | 32915 | + | Hypothetical protein AKS96_48 [Escherichia phage bV_EcoS_AKS96] | 98% | 2e-32 | 96% |
| 45 | 33031 | 33246 | + | Putative holin [Escherichia phage e4/1c] | 98% | 2e-41 | 97% |
| 46 | 33243 | 33728 | + | Endolysin [Escherichia phage bV_EcoS_AHS24] | 98% | 2e-79 | 84% |
| 47 | 33710 | 34114 | + | Putative spanin [Enterobacteria phage vB_EcoS_Rogue1] | 99% | 3e-73 | 94% |
| 48 | 34128 | 34463 | - | Hypothetical protein Rogue1_0065 [Enterobacteria phage vB_EcoS_Rogue1] | 99% | 2e-76 | 100% |
| 49 | 34468 | 36057 | - | Hypothetical protein AHP24_55 [Escherichia phage bV_EcoS_AHP24] | 99% | 0.0 | 99% |
| 50 | 36092 | 36271 | - | Hypothetical protein JK_51 [Escherichia phage Jk06] | 100% | 7e-35 | 98% |
| 51 | 36354 | 36536 | - | Hypothetical protein [Escherichia phage e4/1c] | 98% | 1e-21 | 100% |
| 52 | 36747 | 37100 | - | Hypothetical protein JK_50 [Escherichia phage Jk06] | 99% | 3e-76 | 95% |
| 53 | 37126 | 37428 | - | Hypothetical protein Rogue1_0070 [Enterobacteria phage vB_EcoS_Rogue1] | 99% | 1e-64 | 100% |
| 54 | 37503 | 37655 | - | Hypothetical protein Rogue1_0071 [Enterobacteria phage vB_EcoS_Rogue1] | 80% | 1e-20 | 100% |
| 55 | 37656 | 37895 | - | Hypothetical protein AHS24_64 [Escherichia phage bV_EcoS_AHS24] | 98% | 6e-49 | 97% |
| 56 | 37906 | 38085 | - | Hypothetical protein Rogue1_0073 [Enterobacteria phage vB_EcoS_Rogue1] | 98% | 8e-32 | 97% |
| 57 | 38075 | 38269 | - | Hypothetical protein Rogue1_0074 [Enterobacteria phage vB_EcoS_Rogue1] | 98% | 6e-37 | 100% |
| 58 | 38942 | 39457 | + | Hypothetical protein AHS24_69 [Escherichia phage bV_EcoS_AHS24] | 99% | 8e-122 | 100% |
| 59 | 39457 | 40017 | + | Hypothetical protein AHP24_67 [Escherichia phage bV_EcoS_AHP24] | 99% | 9e-131 | 97% |
| 60 | 40075 | 40305 | + | Hypothetical protein JK_45 [Escherichia phage Jk06] | 98% | 1e-34 | 99% |
| 61 | 40356 | 40589 | + | Hypothetical protein AHP42_66 [Escherichia phage bV_EcoS_AHP42] | 98% | 4e-48 | 100% |
| 62 | 40586 | 40711 | + | Hypothetical protein AKS96_66 [Escherichia phage bV_EcoS_AKS96] | 97% | 6e-19 | 93% |
| 63 | 40708 | 40869 | + | Hypothetical protein [Escherichia phage e4/1c] | 94% | 2e-14 | 61% |
| 64 | 40869 | 41120 | + | Hypothetical protein [Escherichia phage EB49] | 98% | 2e-44 | 84% |
| 65 | 41193 | 41321 | + | Hypothetical protein AHS24_75 [Escherichia phage bV_EcoS_AHS24] | 97% | 3e-20 | 98% |
| 66 | 41318 | 41611 | + | Hypothetical protein JK_42 [Escherichia phage Jk06] | 98% | 1e-63 | 99% |
| 67 | 41615 | 41749 | + | Hypothetical protein AKS96_71 [Escherichia phage bV_EcoS_AKS96] | 97% | 9e-23 | 98% |
| 68 | 41752 | 41991 | + | Putative fusion protein [Escherichia phage bV_EcoS_AHS24] | 98% | 4e-51 | 100% |
| 69 | 41984 | 42178 | + | Hypothetical protein Rogue1_0012 [Enterobacteria phage vB_EcoS_Rogue1] | 98% | 2e-36 | 98% |
| 70 | 42593 | 42742 | + | Hypothetical protein Rogue1_0014 [Enterobacteria phage vB_EcoS_Rogue1] | 98% | 5e-14 | 100% |
| 71 | 42842 | 43357 | + | Putative terminase small subunit [Escherichia phage e4/1c] | 99% | 3e-110 | 98% |
| 72 | 43354 | 44922 | + | Terminase large subunit [Escherichia phage bV_EcoS_AHS24] | 99% | 0.0 | 95% |
| 73 | 44972 | 46246 | + | Portal protein [Escherichia phage bV_EcoS_AKS96] | 99% | 0.0 | 96% |
| 74 | 46227 | 46970 | + | Head morphogenesis protein [Escherichia phage bV_EcoS_AHS24] | 99% | 8e-178 | 99% |
| 75 | 46960 | 47298 | + | Prohead protease [Escherichia phage bV_EcoS_AHS24] | 96% | 2e-70 | 100% |
